# Supplementary material for: The Multifunctionally Graded System for a Controlled Size Effect on Iron Oxide–Gold Based Core-Shell Nanoparticles
Source: Nanomaterials (Basel). 2021 Jun 28;11(7):1695. doi: 10.3390/nano11071695 (PMC8308135; doi:10.3390/nano11071695)
Supplement: Supplementary file 1 [file nanomaterials-11-01695-s001.zip › nanomaterials-1244880-supplementary.pdf]

Supplementary Materials

The Multifunctionally Graded System for a Controlled Size Effect on Iron Oxide–Gold Based Core-Shell Nanoparticles

Bo-Wei Du [1], Chih-Yuan Chu [1], Ching-Chang Lin [2] and Fu-Hsiang Ko [1,*]

[1] Department of Materials Science and Engineering, National Yang Ming Chiao Tung University, Hsinchu 30010, Taiwan; duu.mse04g@nctu.edu.tw (B.-W.D.); dv8962@hotmail.com (C.-Y.C.);

[2] Research Center for Advanced Science and Technology (RCAST), The University of Tokyo, 4-6-1 Komaba, Meguro-ku, Tokyo 153-8904, Japan; Lin@dsc.rcast.u-tokyo.ac.jp

* Correspondence: fhko@mail.nctu.edu.tw; Tel.: +886-35712121 ext: 55803

Table of Contents:

Table S1. The IC50 of Fe_3O_4@Au-dsDNA/DOX for Hela and MCF-7 cells.

Figure S1. Calibration curve of the fluorescence and DOX fluorescence intensity as a function of the DOX and DNA concentration. The plot of log(F_0 − F)/F as a function of log[DNA] was used to calculate the number of bound DOX molecules (*n*) per DNA.

Figure S2. Relationship between the percentage released after applying HFMF for 20 min.

Table S1. The IC50 of Fe_3O_4@Au-dsDNA/DOX for Hela and MCF-7 cells.

Diameter (nm)	IC50-HeLa Cell (μM)	IC50-MCF-7 Cell (μM)
25.9	1.6	1.3
33.1	1.3	1.0
39.5	1.4	1.1
47.7	1.3	1.1
59.5	1.6	1.2

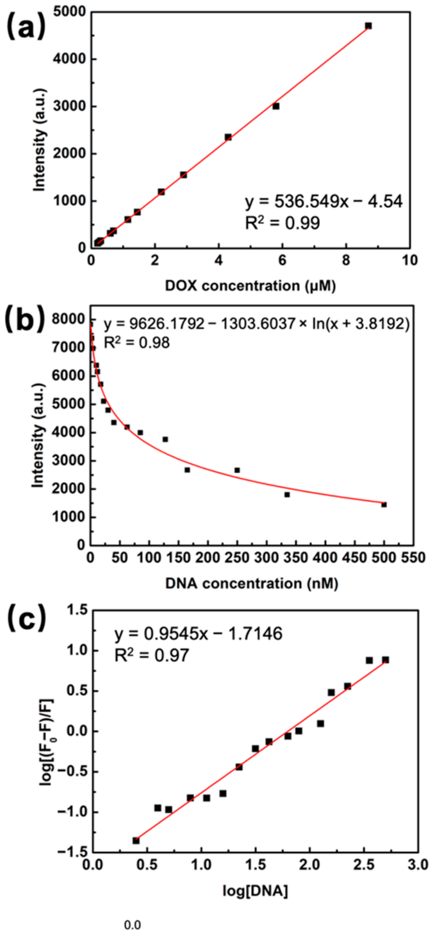

Figure S1. (a) Calibration curve of the fluorescence intensity as a function of the DOX concentration. **(b)** Calibration curve of the DOX fluorescence intensities as a function of the DNA concentrations. **(c)** The plot of $\log(F_0 - F)/F$ as a function of log[DNA] was used to calculate the number of bound DOX molecules (n) per DNA.

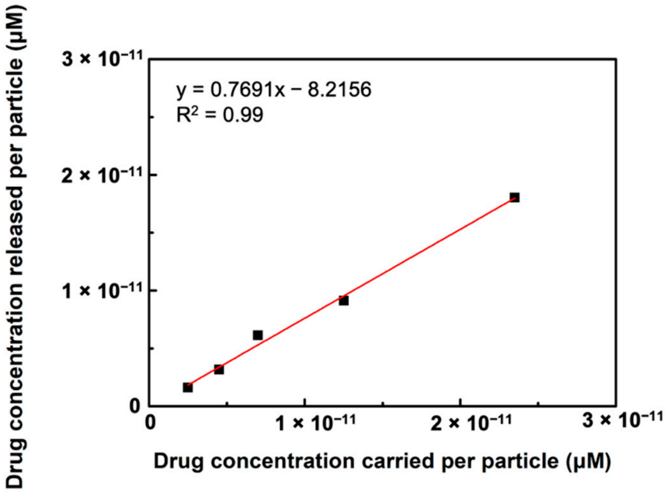

Figure S2. Relationship between the percentage released after applying HFMF for 20 min.
